# Mitochondrial DNA 10609T Promotes Hypoxia-Induced Increase of Intracellular ROS and Is a Risk Factor of High Altitude Polycythemia

**DOI:** 10.1371/journal.pone.0087775

**Published:** 2014-01-30

**Authors:** Chunhua Jiang, Jianhua Cui, Fuyu Liu, Liang Gao, Yongjun Luo, Peng Li, Libin Guan, Yuqi Gao

**Affiliations:** 1 Department of Pathophysiology and High Altitude Physiology, College of High Altitude Military Medicine, Third Military Medical University, Chongqing, PR China; 2 Key Laboratory of High Altitude Medicine, Ministry of Education, Chongqing, PR China; 3 Key Laboratory of High Altitude Medicine, PLA, Chongqing, PR China; 4 Research Center of PLA for Prevention and Treatment of High Mountain Sickness, the 18^th^ Hospital of PLA, Yecheng, Xinjiang, PR China; Vanderbilt University Medical Center, United States of America

## Abstract

Hypobaric hypoxia is the primary cause of high altitude polycythemia (HAPC). Mitochondria are critical organelles that consume high levels of oxygen and generate ATP. We hypothesize that the mitochondrion may be at the center of HAPC, and mitochondrial DNA (mtDNA) SNPs may be involved in its development. First, we conducted a case-control study to investigate the association of mtDNA variants with HAPC in Han Chinese migrating to the Qinghai-Tibetan Plateau. Pearson’s chi-square tests revealed that mtDNA 8414T (MU) frequency (19.5%) in the HAPC group was significantly higher than that of the control (13.0%, P = 0.04, OR = 1.615, 95%CI: 1.020–2.555). The multivariate logistic regression analysis, after adjustment for environmental factors, revealed that mtDNA 10609T (WT) was significantly associated with an increased risk of HAPC (P<0.01, OR = 2.558, 95%CI: 1.250–5.236). Second, to verify the association, *in vitro* experiments of transmitochondrial cybrids was performed and revealed that the mtDNA 10609 variant promoted hypoxia-induced increase of intracellular ROS, but the mtDNA 8414 variant did not. Our findings provide evidence that, in Han Chinese, mtDNA 10609T promotes hypoxia-induced increase of intracellular ROS and is a HAPC risk factor.

## Introduction

High altitude polycythemia (HAPC) is characterized by excessive erythrocytosis (females, Hb ≥19 g/dL; males, Hb ≥21 g/dL) and is encountered in 5 to 18% of the population residing at the Qinghai-Tibetan Plateau [1], [2]. Excessive erythrocytosis leads to a significant increase in blood viscosity, microcirculation disturbance, extensive organ damage, or even death by serious vascular thrombosis [2]. However, there is no effective prevention or treatment for this disease because its pathogenesis is poorly understood. It is a severe public health problem in China and Andean countries because millions of plateau residents may be at risk.

The incidence of HAPC in Europeans is higher than that of the Andes natives, the incidence of HAPC in Andean natives and Han Chinese migrating to the plateau was also significantly higher than that of Tibetans living at the same altitude [3], [4]. In addition, Simonson et al. [5] found that, in Tibetans, hemoglobin concentration was closely related to the single nucleotide polymorphisms (SNP) of several genes. These results suggest that HAPC presents obvious racial and individual differences in susceptibility.

Hypobaric hypoxia is a primary cause of pathophysiological changes at high altitude. The physiologic changes of high altitude acclimatization involve oxygen intake, transportation, and utility [6]. Mitochondria are critical organelles that consume high levels of oxygen [7]. Previous studies have shown that acute hypoxia inhibits the transcription of mitochondrial DNA and damages the structure and function of the mitochondria; however, mitochondrial function can partly recover because of chronic hypoxia [8], [9]. Recently, it was reported that mitochondrial DNA (mtDNA) SNPs affect the activity of the mitochondrial respiratory complex I (NADH dehydrogenase), complex II (succinate dehydrogenase), and complex III (cytochrome c reductase) [10], [11], [12], [13]. In addition, several studies have shown that mtDNA SNPs were not only associated with cancer, high blood pressure, diabetes, but also with human longevity and environmental and climatic adaptability [14], [15], [16]. Recent research has also proven that mtDNA SNPs are associated with acute mountain sickness [17]. These results indicate that the mitochondrion may be at the center of high altitude acclimatization and mountain sickness including HAPC, and that mtDNA SNPs may be involved in HAPC development.

First, we conducted a case-control study to investigate the association between mtDNA SNPs and HAPC in the Han Chinese population. Second, *in vitro* experiments of transmitochondrial cybrids were performed to verify the association.

## Materials and Methods

### Ethics Statement

All subjects were maternally unrelated Han Chinese male volunteers and provided written informed consent for participation in the study, which was approved by the ethics committee of the Third Military Medical University (Chongqing, China).

### Subjects and Procedure

A previous epidemiological study of HAPC in Han Chinese who migrated to the plateau was performed (318 HAPC cases and 881 controls were recruited, data unpublished). 318 HAPC cases and 253 matched controls, continuously exposed to Qinghai-Tibetan Plateau altitudes (3700 m <altitude <5400 m) for more than three months preceding the study were selected. The control group population was similar to the HAPC patients in terms of age, occupation, birthplace, and time spent on the plateau. The subjects aged from 18 to 58 years, from several Chinese provinces, including Gansu province (GS), Shanxi province (SX), Ningxia province (NX), Xinjiang province (XJ), Sichuan province (SC), Hubei province (HB), Chongqing city (CQ), Henan province (HN), Jilin province (JL), Jiangxi province (JX), and Zhejiang province (ZJ), and were born and raised at altitudes lower than 1000 m. Patients with HAPC were diagnosed according to the International Consensus Statement on HAPC [1]. For all subjects, blood samples were drawn into Na-EDTA tubes and stored at −70°C prior to genomic DNA extraction.

In the first phase, we performed demographic investigations and physiological examinations for characteristics analysis of the subjects. To initially screen the mtDNA SNP sites which may be associated with HAPC, we selected 100 participants, composed of 50 patients and 50 matched controls of similar age, occupation, birthplace, and time spent on the plateau for full-length mtDNA sequencing. We then genotyped the screened SNPs in all subjects and analyzed the data to identify the HAPC associated mtDNA SNPs. Finally, we performed the functional studies of transmitochondrial cybrids to verify the association between mtDNA SNPs and HAPC.

### Demographic Investigations and Physiological Examinations

Demographic information was collected by means of a questionnaire (Table S1). Meanwhile, systemic arterial pressure, heart rate, blood oxygen saturation, and weight were measured after a 15-minute rest in the supine position. As described by Jiang et al. [18], assessment of hemoglobin (Hb), plasma erythropoietin (EPO), and plasma reactive oxygen species (ROS) were performed twice at lowland locations by the HiCN method, radioimmunoassay, and fluorometry, respectively.

### Full-length mtDNA Sequencing

The full-length mtDNA sequences of the 100 subjects selected were sequenced bilaterally at the Shanghai Sangon Biological Engineering Technology & Services Co. Ltd., (Shanghai, China). The entire mtDNA sample was amplified into 22 overlapping PCR fragments with the appropriate primers (Table S2). After purification, the amplified DNAs were sequenced, and signals were detected using an ABI 3730 DNA Analyzer (Applied Biosystems, USA). The full-length mtDNA sequences of these subjects have been submitted to the GenBank with accession numbers (KF849892-KF849987). Variations in the mtDNA of the subjects were revealed by comparison with the revised Cambridge Reference Sequence (rCRS). The differences in allele frequency of the mtDNA variants were statistically analyzed, the relationship between each variants allele and Hb or EPO were also analyzed. Therefore, the mtDNA variants potentially associated with HAPC were preliminarily screened.

### SNP Genotyping

Sequencing, polymerase chain reaction restriction fragment length polymorphism (PCR-RFLP), and PCR-high resolution melting analysis (PCR-HRM) were used to genotype the mtDNA variants initially screened (Table S3). Sequencing was carried out according to the methods above. The PCR products in PCR-RFLP were digested with HpaI (New England Biolabs, USA) at 37°C for three hours. The digested products were then separated and visualized on 3% agarose gels stained with ethidium bromide. PCR-HRM was performed in a Rotor-Gene 6000 (Qiagen, Germany) as described by Montgomery et al. [19]. In brief, after general PCR amplification in a 20 µl HRM mixture including Evagreen Dye (Biotium, USA), the amplified DNA was melted, and the high-melting data was analyzed. Finally, thirty percent of the PCR-RFLP and PCR-HRM results were randomly selected and verified by re-sequencing.

### Construction of Transmitochondrial Cybrids

As described by Vithayathil et al. [20], peripheral blood platelets, from HAPC patients (n = 3) with susceptible genotypes and the matched healthy controls (n = 3) with resistant genotypes, were fused into 143B rho0 cells, and thus, changed into transmitochondrial cybrids. Finally, the transmitochondrial cybrids with the susceptible and resistant genotype mtDNA were identified by PCR (The primers were the following: mtDNA-1∶5′-ACACCTATCCCCCATTCTCC-3′ and mtDNA-2∶5′-TGGCTCAGTGT- CAGTTCGAG-3′) and sequencing.

### Measurement of ROS in Cybrids

As described by Ishikawa et al. [12], the intracellular ROS of cybrids under normoxic (21% oxygen) and hypoxic (3% oxygen) conditions was measured. First, the normoxic and hypoxic cybrids were cultured in a normoxic incubator (37°C, 5% carbon dioxide, and 21% oxygen) for 48 hours. Second, hypoxic cybrids were removed from the normoxic incubator and cultured in a hypoxic incubator (37°C, 5% carbon dioxide, and 3% oxygen) for 12 hours. Normoxic cybrids were still cultured in the normoxic incubator for 12 hours. Finally, all of the cybrids were then incubated with 2.5 mM DCFH-DA (Sigma, USA) fluorescent probe at 37°C for 20 minutes, and collected by digestion of trypsin. After washing and suspension with PBS, the average fluorescence intensity of the cybrids was measured by flow cytometry (excitation wave length 488 nm, launch waves 530 nm). The average fluorescence intensity reflected the level of intracellular ROS.

### Quantitative Real-time Polymerase Chain Reaction (qRT-PCR)

Total RNA was isolated from cybrids using Trizol reagent (Invitrogen,USA) according to the Manufacturer’s instructions. Five micrograms of RNA were reverse-transcribed using a reverse transcriptase reaction kit (Life Technologies, USA). Platinum Taq DNA polymerase was purchased from Life Technologies. PCR was performed in triplicate using SYBR Green PCR Master Mix and reactions were carried out on 7500 Fast real-time PCR detection system (Life Technologies, USA) with the amplified conditions: 95°C for 2 min, 40 cycles of 95°C for 10 s, 60°C for 30 s and 72°C for 30 s. The relative expression value was calculated by 2−ΔΔT method. The primer sequences for human beta-actin and EPO were listed in Table S4.

### Statistical Analysis

The Pearson’s chi-square test was used to analyze the demographic and allele frequency distributions in the two groups. Quantitative data are expressed as mean ± standard deviation (mean ± SD) and were compared between cases and controls using unpaired student’s *t*-tests. Multivariate logistic regression analysis was used to analyze the association between mtDNA alleles and HAPC after adjustment for environmental factors. The P-values, odds ratios (ORs), and 95% confidence intervals (95% CI) were calculated. The significance level was set at P<0.05.

## Results

### Subject Characteristics

In total, 318 unrelated HAPC patients and 253 unrelated controls were recruited for the study. The descriptive characteristics of the study population are given in Tables 1 and 2. There was a significant difference in the distribution of migrating altitude between HAPCs and controls (P<0.001); but there was no difference in the distribution of age, smoking, drinking, BMI, or time spent on the plateau (P>0.05), shown in Table 1. The Hb, plasma EPO and ROS content, diastolic pressure, and BMI in the HAPC group were significantly higher than those in the control group (P<0.01), but oxygen saturation was significantly lower than that of the control group (P<0.01). No significant differences were found in the other indices between the two groups (Table 2).

**Table 1:** Characteristics of the study population. (n, %).

Characteristic		Control	HAPC	*p* a
Age	<25 years	135 (56.0%)	166 (58.2%)	0.299
	≥25 years	116 (44.0%)	119 (41.8%)	
Smoking	No	94 (42.3%)	116 (45.8%)	0.443
	Yes	128 (57.7%)	137 (54.2%)	
Drinking	No	193 (88.5%)	226 (89.7%)	0.689
	Yes	25 (11.5%)	26 (10.3%)	
BMI	<24	195 (83.7%)	190 (78.2%)	0.127
	≥24	38 (16.3%)	53 (21.8%)	
Migratingaltitude	<4500 meters	189 (74.7%)	144 (45.3%)	<0.001
	≥4500 meters	64 (25.3%)	174 (54.7%)	
Time onplateau	≤1 years	56 (29.6%)	87 (38.2%)	0.068
	>1 years	133 (70.4%)	141 (61.8%)	

BMI = body mass index;

a = *χ* ^2^ test.

**Table 2:** Assessment of basic physiological indices in the study population.

	Control group		HAPC group	
	n	Mean ± SD	n	Mean ± SD
Hb (g/dL)	253	18.91±1.08	318	22.28±1.27**
Age (years)	251	25.71±6.83	285	25.25±6.30
EPO (ng/ml)	230	1.46±0.51	246	1.62±0.58**
ROS (fluorescence intensity)	86	540.36±134.96	114	591.44±130.25*
Systolic pressure (mmHg)	234	123.07±15.13	241	124.58±12.74
Diastolic pressure (mmHg)	234	74.90±11.55	241	77.89±11.10**
Blood oxygen saturation (%)	234	89.55±3.56	243	87.86±3.55**
Heart rate (time/minute)	234	84.21±14.04	243	85.95±14.30
BMI	233	21.47±2.45	254	22.14±2.44**
Time on plateau (months)	189	39.83±46.10	228	35.01±37.97

Data are presented as mean ± SD. Hb = hemoglobin; EPO = erythropoietin; ROS = reactive oxygen species; BMI = body mass index.

* compared with the control group = P<0.05;

** compared with the control group = P<0.01.

### Initial Screening of Potential HAPC Associated mtDNA Variants

Sequencing of the full-length mtDNA sequences was completed for 96 of the 100 samples selected. The full-length mtDNA sequences of remaining four samples can not be sequenced bilaterally because of gathering of ‘C’ base, and thus the remaining four samples were excluded. After comparison with the rCRS, 68 mtDNA variants with a frequency greater than 5% were observed in the coding region (Table S5). The comparison between the wild and mutant genotypes of the above mtDNA variants indicated that 16 mtDNA variants were associated with Hb and plasma EPO, suggesting that these variants may be associated with HAPC, as shown in Table 3.

**Table 3:** Initial screening of potential HAPC associated mtDNA variants.

MtDNAvariants	Location	Amino acidchange	Relatedfactors	*p* a
A663G	12SrRNA	–	EPO	0.045
A1736G	16SrRNA	–		
G3010A	16SrRNA	–	Hb	0.024
C8414T	ATP8	L>F	Hb	0.018
C14668T	ND6	–		
A8701G	ATP6	T>A	Hb	0.006
C10400T	ND3	T>A		
T10873C	ND4	–		
T14783C	CYTB	–		
G15043A	CYTB	–		
G9053A	ATP6	S>N	Hb	0.028
A10398G	ND3	T>A	Hb	0.006
T10609C	ND4l	M>T	Hb	0.019
G12406A	ND5	V>I		
G11696A	ND4	V>I	Hb	0.005
G15301A	CYTB	–	Hb	0.004

Hb = hemoglobin; EPO = erythropoietin;

a = *t* test.

### Correlation of mtDNA Variants and HAPC Susceptibility

The chi-square test results showed that the frequency of mtDNA 8414T in the HAPC group (19.5%) was significantly higher than that of the control group (13.0%, P = 0.04, OR = 1.615, 95% CI: 1.020–2.555). There was no significant difference in the rest of the allele frequencies of the other variants between the two groups (Table 4).

**Table 4:** Allele frequency of mtDNA variants in the HAPC and control groups (%).

Site	Genotype	HAPC group	Control group	HAPC Vs. Control		
				OR	95% CI	*P* value
A663G	G	9.1% (29/318)	9.5% (24/253)	0.957	0.543–1.690	0.881
A1736G	G	8.2% (26/318)	7.1% (18/253)	1.162	0.622–2.172	0.637
G3010A	A	17.9% (57/318)	13.8% (35/253)	1.360	0.861–2.150	0.187
C8414T	T	19.5% (62/318)	13.0% (33/253)	1.615	1.020–2.555	0.040*
A8701G	G	47.5% (151/318)	46.6% (118/253)	1.034	0.743–1.440	0.841
G9053A	A	9.1% (29/318)	11.1% (28/253)	0.806	0.466–1.394	0.441
A10398G	G	57.9% (184/318)	55.7% (141/253)	1.091	0.782–1.522	0.610
C10400T	T	51.6% (164/318)	49.4% (125/253)	1.090	0.784–1.517	0.607
T10609C	C	10.1% (32/318)	15.4% (39/253)	0.614	0.372–1.012	0.054
T10873C	C	50.3% (160/318)	47.4% (120/253)	1.122	0.807–1.562	0.494
G11696A	A	2.8% (9/318)	2.4% (6/253)	1.199	0.421–3.414	0.734
G12406A	A	11.3% (36/318)	15.8% (40/253)	0.680	0.419–1.103	0.117
C14668T	T	17.3% (55/318)	13.0% (33/253)	1.394	0.874–2.225	0.162
T14783C	C	51.6% (164/318)	49.4% (125/253)	1.090	0.784–1.517	0.607
G15043A	A	51.9% (165/318)	49.8% (126/253)	1.087	0.781–1.512	0.621
G15301A	A	52.8% (168/318)	49.0% (124/253)	1.165	0.837–1.622	0.365

* compared with the control group = P<0.05.

Multivariate logistic regression analysis revealed that, after adjustment for factors such as age, BMI, smoking, drinking, time spent on the plateau, and migrating altitude, mtDNA 10609C (MU) was associated with a 0.391-fold decreased risk of developing HAPC (P<0.01,OR = 0.391, 95%CI: 0.191–0.800). Conversely, mtDNA 10609T (WT) was associated with a 2.5-fold increased risk of developing HAPC (P<0.01, OR = 2.558, 95% CI: 1.250–5.236). However, after adjustment for environmental factors, the mutant genotype of the mtDNA 8414 site and the remaining fourteen mtDNA variants did not affect the risk of developing HAPC for Han Chinese migrating to the plateau (Table 5). The above results show that the mtDNA 8414T (MU) and mtDNA 10609T (WT) are risk factors for developing HAPC in the Han Chinese population studied.

**Table 5:** Multiple logistic regression analysis of the relationship between HAPC and mtDNA variants, adjusted for environmental factors.

Variable	OR	95% CI	*P* value^a^
mtDNA 663G	1.227	0.596–2.526	0.579
mtDNA 1736G	1.629	0.754–3.520	0.214
mtDNA 3010A	0.723	0.395–1.323	0.293
mtDNA 8414T	0.938	0.521–1.689	0.831
mtDNA 8701G	1.103	0.713–1.707	0.659
mtDNA 9053A	0.457	0.202–1.036	0.061
mtDNA 10398G	1.167	0.750–1.816	0.494
mtDNA 10400T	1.13	0.729–1.750	0.585
mtDNA 10609C	0.391	0.191–0.800	<0.01
mtDNA 10873C	1.191	0.770–1.843	0.431
mtDNA 11696A	0.858	0.215–3.431	0.829
mtDNA 12406A	0.511	0.259–1.006	0.052
mtDNA 14668T	0.805	0.436–1.486	0.489
mtDNA 14783C	1.139	0.735–1.765	0.561
mtDNA 15043A	1.147	0.739–1.778	0.541
mtDNA 15301A	1.206	0.778–1.870	0.402

OR = odds ratio; CI = confidence interval.

a = WT genotype is ref.

### Identification of the Transmitochondrial Cybrids

**Figure 1:**
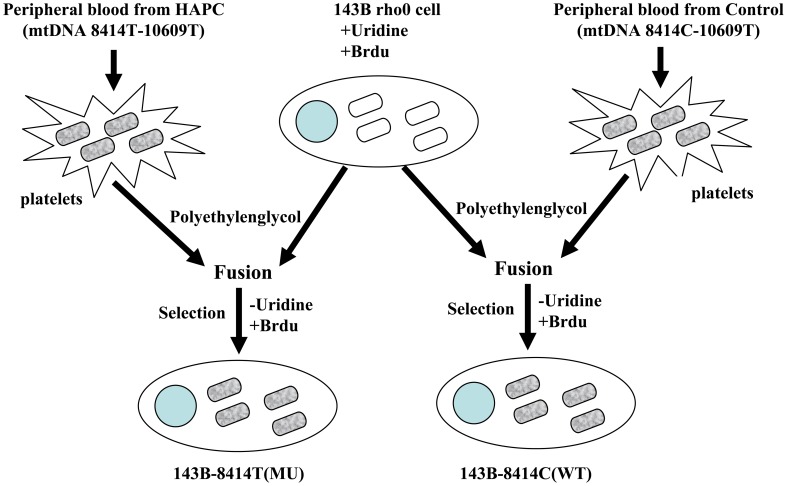
Schematic of transmitochondrial cybrid generation for the mtDNA 8414 site.

**Figure 2:**
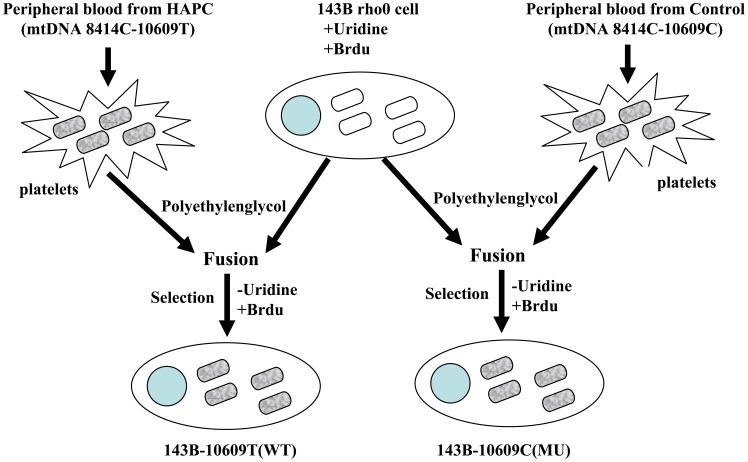
Schematic of transmitochondrial cybrid generation for the mtDNA 10609 site.

**Figure 3:**
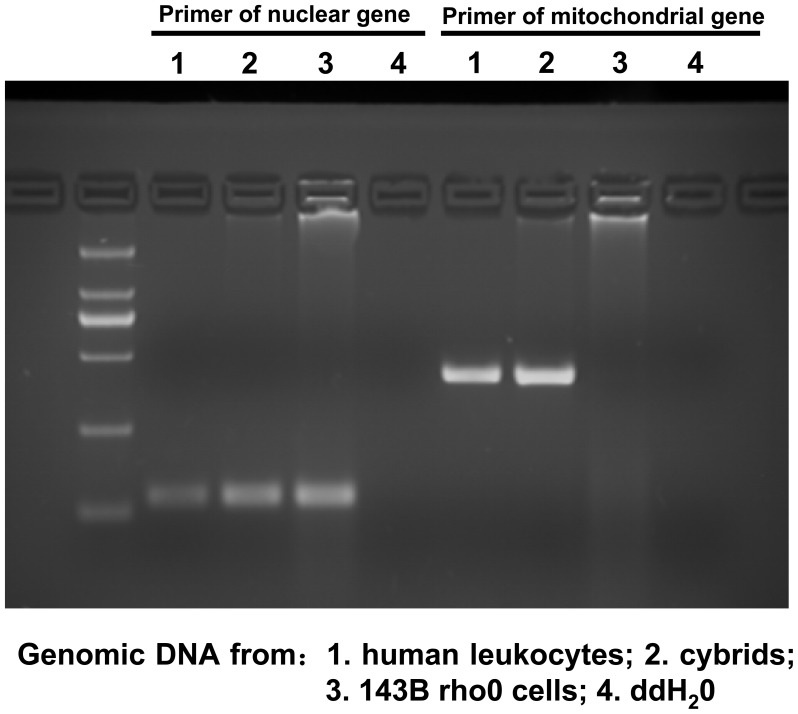
Identification of the transmitochondrial cybrids by PCR.

According to the correlation analysis results, platelets from corresponding patients and matched controls were fused to the 143B rho0 cells. The fusion cells were then cultured in a medium with bromodeoxyuridine and without pyruvate (Figs. 1 and 2). PCR results revealed that the 143B rho0 cells were unable to amplify the mtDNA PCR products, but the rho0 cells fusing with platelets could (Fig. 3). At the same time, the mtDNA sequence of the fusion cells was consistent with that of the platelet provider, indicating successful fusion between mitochondria and 143B rho0 cells.

### Effect of mtDNA C8414 T and T10609C Polymorphisms on Intracellular ROS

**Figure 4:**
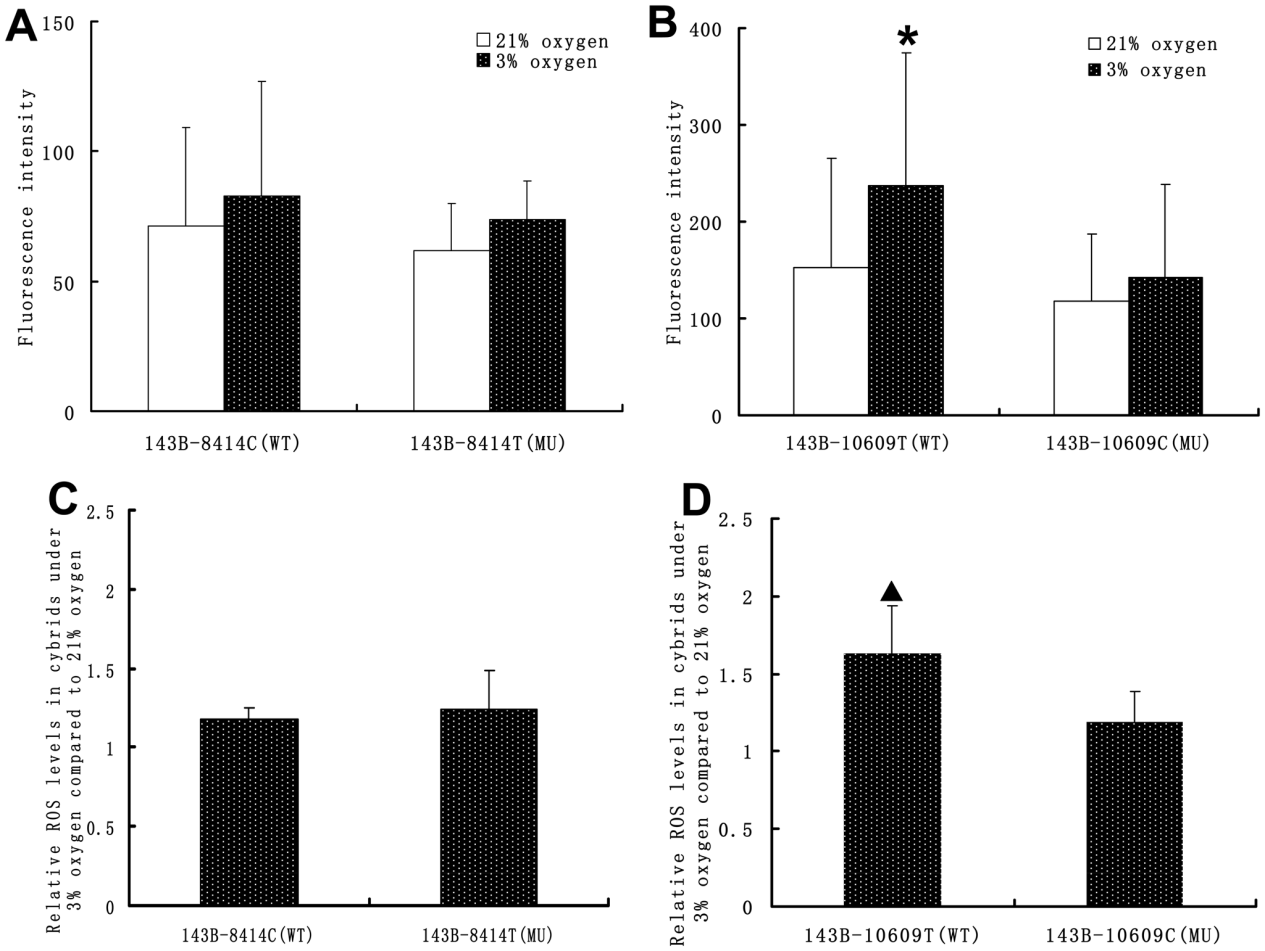
Intracellular ROS in the cybrids under normoxic and hypoxic conditions. First, the normoxic and hypoxic cybrids were cultured in a normoxic incubator (37°C, 5% carbon dioxide, and 21% oxygen) for 48 hours. Second, hypoxic cybrids were shifted and cultured in a hypoxic incubator (37°C, 5% carbon dioxide, and 3% oxygen) for 12 hours. Normoxic cybrids were still cultured in the normoxic incubator for 12 hours. Finally, all of the cybrids were incubated with 2.5 mM DCFH-DA fluorescent probe. The average fluorescence intensity of the cybrids was measured by flow cytometry. The average fluorescence intensity reflected the level of intracellular ROS. All cultures were done in triplicate (n = 3). A. Intracellular ROS in 143B-8414C (WT) and 143B-8414T (MU) under normoxic and hypoxic conditions. B. Intracellular ROS in 143B-10609T (WT) and 143B-10609C (MU) under normoxic and hypoxic conditions. C. The increasing rate of intracellular ROS relative to normoxic conditions in the 143B-8414C (WT) and 143B-8414T (MU) cybrids. D. The increasing rate of intracellular ROS relative to normoxic conditions in the 143B-10609T (WT) and 143B-10609C (MU) cybrids. Error bars indicate the standard deviation. *P<0.05; significantly different from culture under 21% oxygen. ▴ P<0.05; significantly different from 143B-10609C (MU) cybrids.

There was no significant difference in intracellular ROS between the 143B-8414C (WT) and 143B-8414T (MU) cybrids under normoxic conditions, or between the 143B-10609 (WT) and 143B-10609C (MU) cybrids. In comparison with normoxic conditions, there was a significant increase in intracellular ROS in the 143B-10609T (WT) cybrids (P<0.05), but not in 143B-8414C (WT), 143B-8414T (MU), or 143B-10609C (MU) cybrids under hypoxic conditions. The increasing rate of intracellular ROS relative to normoxic conditions in the 143B-10609T (WT) cybrids was significantly higher than that in the 143B-10609C (MU) cybrids, but no significant difference was observed between the 143B-8414C (WT) and 143B-8414T (MU) cybrids (Fig. 4).

### The mRNA Level of EPO in Cybrids

**Figure 5:**
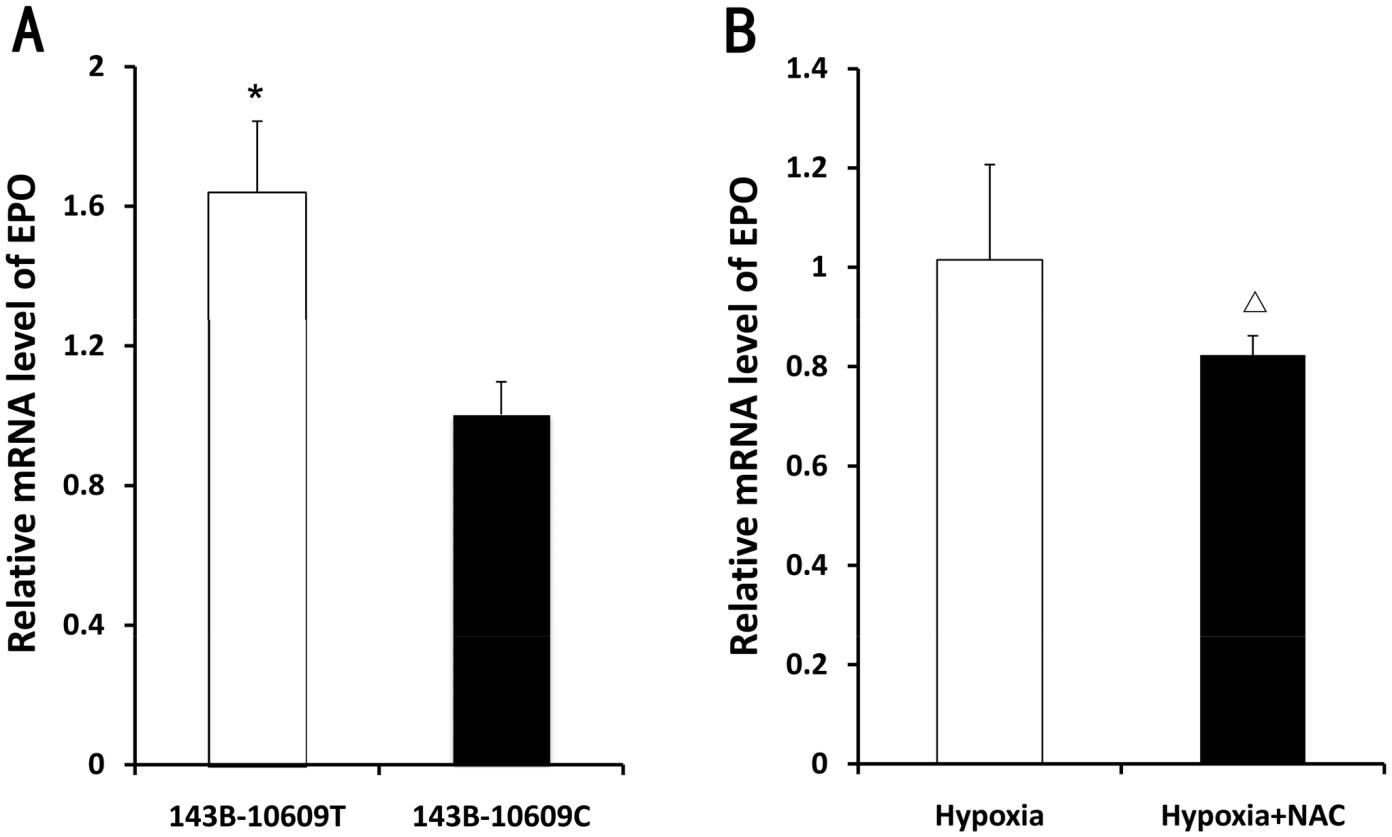
The mRNA level of EPO in cybrids. A. *qRT-PCR* analysis of mRNA level of EPO from 143B-10609T (WT) and 143B-10609C (MU) cultured for 12 h under hypoxic conditions (37°C, 5% carbon dioxide, and 3% oxygen) (n = 3). B. *qRT-PCR* analysis of mRNA level of EPO from 143B-10609T (WT) incubated in the absence or presence of 2.5 mM N-acetyl-L-cysteine (NAC) for 12 h under hypoxic conditions (37°C, 5% carbon dioxide, and 3% oxygen) (n = 3). Housekeeping gene was beta-actin, and the relative expression value was calculated by 2−ΔΔT method. Error bars indicate the standard deviation. * P<0.01; significantly different from 143B-10609C (MU). ΔP<0.05; significantly different from 143B-10609T (WT) incubated in the absence of 2.5 mM NAC.

The mRNA level of EPO in the 143B-10609T (WT) was significantly higher than that in 143B-10609C (MU) under hypoxic condition (P<0.01). Under hypoxic condition, treatment with 2.5 mM N-acetyl-L-cysteine (NAC) for 12 h caused significant decrease in the mRNA level in the 143B-10609T (WT) (P<0.05) (Fig. 5).

## Discussion

Previous epidemiological investigations of HAPC in Han Chinese migrating to the Qinghai-Tibetan Plateau have demonstrated that HAPC is significantly correlated with the age of the subjects, time spent on the plateau, and labor intensity [3], [21], [22]. An additional investigation in our lab has revealed similar results (unpublished data). In addition, obvious differences in the incidences of HAPC have been reported between different races, such as between Tibetans, Han Chinese, and Andean natives [3], [4], [23]. These outcomes indicate a complex pathogenesis in HAPC, resulting from the interaction of environmental and genetic factors. In this study, demographic investigations show that there is no difference in the distributions of age, time spent on the plateau, smoking and drinking habits between the control and HAPC groups, suggesting a good match between the two groups, and providing a basis for future genetic analysis.

The characteristics of the study population show that the BMI in the HAPC group is significantly higher than those in the control group (P<0.01). We presume that people with higher BMI is relatively heavy, consuming more oxygen, enduring more serious hypoxia at same plateau, and therefore is more susceptible to HAPC, which requires further study. There is tendency for higher blood pressure in HAPCs because of increased blood viscosity, which is in contrast to the patients with Chuvash polycythemia [2], [24].

Takasaki et al. [25] reported that the mtDNA 8414 variant is correlated with longevity, and that persons having mtDNA 8414T have a high probability of living to the age of 100. The variation of mtDNA8414 C>T (L>F) is a missense mutation and may have an effect on its locus gene–ATP synthase. In this study, the frequency of mtDNA 8414T (19.5%) in the HAPC group is significantly higher than that of the control group (13.0%), suggesting that this variant is a risk factor for developing HAPC for the Han population migrating to the plateau, and that the ATP synthase subunit 8 in the mitochondria may be the HAPC susceptible gene.

The mitochondrial respiratory complex, whose activity is influenced by mitochondrial DNA [10], [11], is the main source of ROS production. The ROS catabolic enzymes, including manganese superoxide dismutase (MnSOD) and glutathione (GSH)-dependent peroxidase enzyme (GSH/Px), mainly located the cytoplasm and mitochondrial matrix, ensures that intracellular ROS are kept at a nontoxic level [26]. The 143B-8414C (WT) and 143B-8414T (MU) cybrids have the same nucleus, cytoplasm, and mitochondrial matrix, but not mitochondrial DNA. Accordingly, the difference in intracellular ROS between these cybrids is determined by mtDNA. The results of the *in vitro* experiments in the transmitochondrial cybrids showed no significant differences in intracellular ROS between 143B-8414C (WT) and 143B-8414T (MU) cybrids, regardless of whether they were cultured normoxic or hypoxic conditions. This indicates that the mtDNA 8414 variant has no effect on the activity of the mitochondrial respiratory complex and intracellular ROS.

The mtDNA 10609 variant is a common mtSNP. To our knowledge, there is no report on the association between the mtDNA 10609 variant and disease. In this study, logistic regression analysis revealed that mtDNA 10609C (MU) significantly reduced risk for developing HAPC and conversely, mtDNA 10609T (WT) significantly increased them. This indicates that mtDNA 10609 variant is closely related to HAPC susceptibility in the Han population who migrated to the plateau area, and the gene-ND4L in which the mtDNA 10609 site is located is the HAPC susceptibility gene in this population. The *in vitro* experimental results on mitochondrial function in the cybrids show that, there is no difference in the intracellular ROS between 143B-10609T (WT) and 143B-10609C (MU). However, after hypoxic exposure (3% oxygen, 12 hours), 143B-10609T (WT) intracellular ROS significantly increases, but this is not the case in 143B-10609C (MU). This suggests that the mtDNA 10609 polymorphism can affect mitochondrial function, and the cells carrying mtDNA 10609T (WT) can generate more ROS under hypoxic conditions.

The mitochondrial respiratory chain complex I is the first portal of the respiratory chain to generate ROS [26]. ND4L is a subunit of the mitochondrial respiratory chain complex I, and is required for the normal assembly and activity of mitochondrial complex I [27]. The main function of the respiratory chain complex I is to transmit two oxygen electrons from the NADH of carbohydrates, fats, and proteins to the ubiquinone binding with the complex I membrane area [28], [29]. Four protons are then transferred from the matrix to the inner mitochondrial membrane producing a cross membrane proton electrochemical gradient and induces the formation of ROS [30]. MtDNA 10609 T → C is a missense mutation, changing the polar ND4L 47 amino acids into non-polar methionine causing changes in the ND4L protein sequence, which may affect the function of mitochondrial respiratory chain complex I and the production of ROS coming from mitochondrion under hypoxic conditions. In addition, the results of the plasma ROS assessments showed that the plasma ROS in the HAPC group was significantly higher than those in the control group. These results indicate that ROS may be involved in the development of HAPC.

Studies have shown that ROS can increase hypoxia-inducible factor −1α (HIF-1α) protein levels by inhibiting HIF-1α proline hydroxylase activity [31], [32], [33]. This is an important mechanism for regulating HIF-1α protein levels in the cells. HIF-1 activity depends mainly on the protein levels of the α subunit [34]. The synthesis and release of EPO is regulated by a variety of factors, such as HIF-1 increased by inadequate tissue oxygen. The expression of EPO is promoted through the binding of HIF-1 to the hypoxia-response element [35]. In this study, the mRNA level of EPO in the 143B-10609T (WT) was higher than that in the 143B-10609C (MU) under hypoxic condition. At the same time, under hypoxic condition, removal of ROS reduced the mRNA level of EPO in the cybrids (143B-10609T), which is consistent with the reports of Chandel et al. [36]. Thus, ROS contributes to the expression of downstream target genes such as EPO. It is generally believed that enhanced stimulation of bone marrow erythropoiesis by EPO is a key factor in HAPC pathogenesis [35], [37], [38]. In this study, the plasma EPO of HAPC group was significantly higher than that of the control group, which is consistent with the literature.

To sum up, our findings indicate that mtDNA 10609T promotes hypoxia-induced increase of intracellular ROS and increases the risk of developing HAPC via the ROS-HIF-EPO pathway, which requires further study.

## Supporting Information

Table S1 Questionnaire for plateau residents.
(DOC)

Table S2 Primers for mitochondrial genomic DNA sequencing.
(DOC)

Table S3 Primer sequences and PCR conditions for mitochondrial DNA variant genotyping.
(DOC)

Table S4 Primer sequences and PCR conditions for beta-actin and EPO.
(DOC)

Table S5 MtDNA variant allele frequencies in the 47 controls and 49 HAPC patients.
(DOC)
