# Supplementary material for: Mitochondrial DNA 10609T Promotes Hypoxia-Induced Increase of Intracellular ROS and Is a Risk Factor of High Altitude Polycythemia
Source: PLoS One. 2014 Jan 30;9(1):e87775. doi: 10.1371/journal.pone.0087775 (PMC3907523; doi:10.1371/journal.pone.0087775)
Supplement: Table S1 — Questionnaire for plateau residents. (DOC) [file pone.0087775.s001.doc]

Table S1.

Questionnaire for plateau residents

| Name |  | | Gender |  | | | Birthday | | yy mm dd | | | | |
| --- | --- | --- | --- | --- | --- | --- | --- | --- | --- | --- | --- | --- | --- |
| Nationality |  | | Affiliation |  | | | | Occupation | | | |  | |
| Location |  | | Altitude | m | | | Birthplace | |  | | | | |
| Ancestral home | | province city | | | | | Telephone | |  | | | | |
| Smoking | yes or no | | Drinking | yes or no | | | The number of reach plateau | | | | | |  |
| The first day on the plateau | | | yy mm | | The latest day reach plateau | | | | | | yy mm | | |
| Time on plateau | | (months) | | The previous living plateau | | | | |  | | | | |
| High altitude disease history | | | AMS（yes or no） HAPE（yes or no） HACE（yes or no） | | | | | | | | | | |
| Other diseases | | no or yes（ ） | | diploma | middle school, high school, university | | | | | | | | |
| Symptom at rest | | | | | | | | | | | | | |
| Breathlessness and/or palpitations | | 0 No（ ）；1 Mild（ ）；2 Moderate（ ）；3 Severe（ ） | | | | | | | | | | | |
| Sleep disturbance | | 0 Slept as well as usual（ ）；1 Did not sleep as well as usual（ ）；2 Woke many times, poor night’s sleep（ ）；3 Could not sleep at all（ ） | | | | | | | | | | | |
| Cyanosis | | 0 No（ ）；1 Mild（ ）；2 Moderate（ ）；3 Severe（ ） | | | | | | | | | | | |
| Dilatation of veins | | 0 No（ ）；1 Mild（ ）；2 Moderate（ ）；3 Severe（ ） | | | | | | | | | | | |
| Paresthesia | | 0 No（ ）；1 Mild（ ）；2 Moderate（ ）；3 Severe（ ） | | | | | | | | | | | |
| Headache | | 0 No（ ）；1 Mild（ ）；2 Moderate（ ）；3 Severe（ ） | | | | | | | | | | | |
| Tinnitus | | 0 No（ ）；1 Mild（ ）；2 Moderate（ ）；3 Severe（ ） | | | | | | | | | | | |
| Hb | | g/dL; Score: | | | | CMS Score | | | |  | | | |
