# Supplementary material for: Mitochondrial DNA 10609T Promotes Hypoxia-Induced Increase of Intracellular ROS and Is a Risk Factor of High Altitude Polycythemia
Source: PLoS One. 2014 Jan 30;9(1):e87775. doi: 10.1371/journal.pone.0087775 (PMC3907523; doi:10.1371/journal.pone.0087775)
Supplement: Table S2 — Primers for mitochondrial genomic DNA sequencing. (DOC) [file pone.0087775.s002.doc]

Table S2.

Primers for mitochondrial genomic DNA sequencing

| **PCR Fragment** | **No.** | **Starting position** | Primer sequence (5'-3') |
| --- | --- | --- | --- |
| Fragment 1 | 1 | hmtL569 | F: AACCAAACCCCAAAGACACC |
|  | 2 | hmtL1315 | F: GTAAGCGCAAGTACCCACG |
|  | 3 | hmtH1405 | R: ATCCACCTTCGACCCTTAAG |
|  | 4 | hmtL2070 | F: AATTTGCCCACAGAACCCTC |
|  | 5 | hmtH2174 | R: ATTGGTGGCTGCTTTTAGGC |
|  | 6 | hmtH2941 | R: GACTCTAGAATAGGATTGCGC |
| Fragment 2 | 7 | hmtL2797 | F: GTCCTAAACTACCAAACCTGC |
|  | 8 | hmtL3568 | F: CGCTCTTCTACTATGAACCC |
|  | 9 | hmtH3733 | R: ATGATGGCTAGGGTGACTTC |
|  | 10 | hmtL4322 | F: ATAATAGGAGCTTAAACCCCC |
|  | 11 | hmtH4501 | R: TGTGCCTGCAAAGATGGTAG |
|  | 12 | hmtH5193 | R: GTGTTAGTCATGTTAGCTTG |
| Fragment 3 | 13 | hmtL5061 | F: AGCAGTTCTACCGTACAACC |
|  | 14 | hmtL5828 | F: GAAAATCACCTCGGAGCTGG |
|  | 15 | hmtH5993 | R: TAAGGAGGCTTAGAGCTGTG |
|  | 16 | hmtL6563 | F: ACCTCAACACCACCTTCTTC |
|  | 17 | hmtH6753 | R: TGTGCTCACACGATAAACCC |
|  | 18 | hmtH7497 | R: TTTGAAAAAGTCATGGAGGCC |
| Fragment 4 | 19 | hmtL7336 | F: GATTTGAGAAGCCTTCGCTTC |
|  | 20 | hmtL8080 | F: TCTTGCACTCATGAGCTGTC |
|  | 21 | hmtH8251 | R: GCTATAGGGTAAATACGGGC |
|  | 22 | hmtL8815 | F: CTCATTTACACCAACCACCC |
|  | 23 | hmtH9030 | R: CCAATTAGGTGCATGAGTAGG |
|  | 24 | hmtH9819 | R: GCCAATAATGACGTGAAGTCC |
| Fragment 5 | 25 | hmtL9611 | F: TCCCACTCCTAAACACATCC |
|  | 26 | hmtL10380 | F: TCTGGCCTATGAGTGACTAC |
|  | 27 | hmtH10598 | R: GTTGAGGGTTATGAGAGTAGC |
|  | 28 | hmtL11107 | F: TTCACAGCCACAGAACTAATC |
|  | 29 | hmtH11381 | R: AAGTGGAGTCCGTAAAGAGG |
|  | 30 | hmtH12111 | R: AAACCCGGTAATGATGTCGG |
| Fragment 6 | 31 | hmtL11727 | F: GCCCACGGGCTTACATC |
|  | 32 | hmtL12640 | F: TCGTTACATGGTCCATCATAG |
|  | 33 | hmtH12862 | R: AAACCGATATCGCCGATACG |
|  | 34 | hmtL13389 | F: TCCATCATCCACAACCTTAAC |
|  | 35 | hmtH13627 | R: AAGCGAGGTTGACCTGTTAG |
|  | 36 | hmtH14559 | R: GATTGTTAGCGGTGTGGTCG |
| Fragment 7 | 37 | hmtL14130 | F: TCTTCCCACTCATCCTAACC |
|  | 38 | hmtL14838 | F: TCCAACATCTCCGCATGATG |
|  | 39 | hmtH15153 | R: CCCCTCAGAATGATATTTGGC |
|  | 40 | hmtH15912 | R: TCTCCGGTTTACAAGACTGG |
| Fragment 8 | 41 | hmtL15591 | F: TTCGCCTACACAATTCTCCG |
|  | 42 | hmtL16365 | F: GTCAAATCCCTTCTCGTCCC |
|  | 43 | hmtH112 | R: ACAGATACTGCGACATAGGG |
|  | 44 | hmtH626 | R: TTTATGGGGTGATGTGAGCC |
