# Supplementary material for: Mitochondrial DNA 10609T Promotes Hypoxia-Induced Increase of Intracellular ROS and Is a Risk Factor of High Altitude Polycythemia
Source: PLoS One. 2014 Jan 30;9(1):e87775. doi: 10.1371/journal.pone.0087775 (PMC3907523; doi:10.1371/journal.pone.0087775)
Supplement: Table S3 — Primer sequences and PCR conditions for mitochondrial DNA variant genotyping. (DOC) [file pone.0087775.s003.doc]

Table S3.

Primer sequences and PCR conditions for mitochondrial DNA variant genotyping

| **Site** | **Method** | **Primer sequence (5'-3')** | **PCR region** | **Annealing temperature** | **Length of PCR product** |
| --- | --- | --- | --- | --- | --- |
| A663G | PCR-HRM | F:TGAAAATGTTTAGACGGGCTC | 610-703 | 57ºC | 94 bp |
|  |  | R:TGCTTGCATGTGTAATCTTAC |  |  |  |
| A1736G | PCR-HRM | F:GCTAAACCTAGCCCCAAACC | 1671-1776 | 60ºC | 106 bp |
|  |  | R:CTATTGCGCCAGGTTTCAAT |  |  |  |
| G3010A | PCR-HRM | F:GGATAACAGCGCAATCCTAT | 2933-3049 | 57ºC | 117 bp |
|  |  | R:ATCGTTGAACAAACGAACCT |  |  |  |
| C8414T | PCR-HRM | F:TCTTTACAGTGAAATGCCCC | 8353-8472 | 58ºC | 120 bp |
|  |  | R:GGTAGGTGGTAGTTTGTGTT |  |  |  |
| A8701G | PCR-HRM | F:CCGACTAATCACCACCCAAC | 8646-8730 | 60ºC | 85 bp |
|  |  | R:TCAGGTTCGTCCTTTAGTGTTGT |  |  |  |
| G9053A | PCR-HRM | F:ACCGCTAACATTACTGCAGG | 9007-9076 | 60ºC | 70 bp |
|  |  | R:TGGTTGATATTGCTAGGGTGG |  |  |  |
| A10398G  C10400T  T10609C  T10873C | Sequencing |  |  |  |  |
| G11696A | PCR-HRM | F:CAGCCATTCTCATCCAAACC | 11655-11778 | 60ºC | 124 bp |
|  |  | R:CGACTGTGAGTGCGTTCGTA |  |  |  |
| G12406A | PCR-RFLP | F:AAGAACTGCTAACTCATGCC | 12219-12631 | 58ºC | 413 bp |
|  |  | R:ACCATGTAACGAACAATGCT |  |  |  |
| C14668T  T14783C  G15043A | Sequencing |  |  |  |  |
| G15301A | PCR-HRM | F:AGACAGTCCCACCCTCACAC | 15256-15337 | 60ºC | 82 bp |
|  |  | R:GAGGTGGAGTGTTGCTAGGG |  |  |  |
