# Supplementary material for: Mitochondrial DNA 10609T Promotes Hypoxia-Induced Increase of Intracellular ROS and Is a Risk Factor of High Altitude Polycythemia
Source: PLoS One. 2014 Jan 30;9(1):e87775. doi: 10.1371/journal.pone.0087775 (PMC3907523; doi:10.1371/journal.pone.0087775)
Supplement: Table S4 — Primer sequences and PCR conditions for beta-actin and EPO. (DOC) [file pone.0087775.s004.doc]

Table S4.

Primer sequences and PCR conditions for beta-actin and EPO

| **Gene** | **Primer sequence (5'-3')** | **Annealing temperature** |
| --- | --- | --- |
| Beta-actin | F: CTCTGGCCGTACCACTGGC | 60ºC |
|  | R: GTGAAGCTGTAGCCGCGC |  |
| Erythropoietin | F: CAGTGATTGTTCGGAGTGGAGC | 60ºC |
|  | R: TGCATGTGGATAAAGCCGTCAG |  |
