# Supplementary material for: Mitochondrial DNA 10609T Promotes Hypoxia-Induced Increase of Intracellular ROS and Is a Risk Factor of High Altitude Polycythemia
Source: PLoS One. 2014 Jan 30;9(1):e87775. doi: 10.1371/journal.pone.0087775 (PMC3907523; doi:10.1371/journal.pone.0087775)
Supplement: Table S5 — MtDNA variant allele frequencies in the 47 controls and 49 HAPC patients. (DOC) [file pone.0087775.s005.doc]

Table S5.

MtDNA variant allele frequencies in the 47 controls and 49 HAPC patients (n, %)

| **Site** | **Local gene** | **Genotype** | **Amino acids** | **Allele frequency** | | | **P-value** |
| --- | --- | --- | --- | --- | --- | --- | --- |
|  |  |  |  | **all**  **(n＝96)** | **control**  **(n＝47)** | **HAPC**  **(n＝49)** |  |
| A663G | 12S rRNA | G |  | 6.3% | 8.5% (4/47) | 4.1% (2/49) | ＞0.05 |
| G709A | 12S rRNA | A |  | 17.7% | 14.9% (7/47) | 20.4% (10/49) | ＞0.05 |
| C752T | 12S rRNA | T |  | 6.3% | 6.4% (3/47) | 6.1% (3/49) | ＞0.05 |
| T1005C | 12S rRNA | C |  | 5.2% | 6.4% (3/47) | 4.1% (2/49) | ＞0.05 |
| T1107C | 12S rRNA | C |  | 8.3% | 8.5% (4/47) | 8.2% (4/49) | ＞0.05 |
| A1438G | 12S rRNA | G |  | 93.8% | 93.6% (44/47) | 93.9% (46/49) | ＞0.05 |
| A1736G | 16S rRNA | G |  | 6.3% | 8.5% (4/47) | 4.1% (2/49) | ＞0.05 |
| T1824C | 16S rRNA | C |  | 5.2% | 6.4% (3/47) | 4.1% (2/49) | ＞0.05 |
| G3010A | 16S rRNA | A |  | 15.6% | 19.1% (9/47) | 12.2% (6/49) | ＞0.05 |
| T3394C | ND1 | C | Y-H | 5.2% | 2.1% (1/47) | 8.2% (4/49) | ＞0.05 |
| C3970T | ND1 | T |  | 19.8% | 21.3% (10/47) | 18.4% (9/49) | ＞0.05 |
| C4071T | ND1 | T |  | 7.3% | 6.4% (3/47) | 8.2% (4/49) | ＞0.05 |
| C4086T | ND1 | T |  | 7.3% | 10.6% (5/47) | 4.1% (2/49) | ＞0.05 |
| T4248C | ND1 | C |  | 5.2% | 6.4% (3/47) | 4.1% (2/49) | ＞0.05 |
| A4715G | ND2 | G |  | 12.5% | 10.6% (5/47) | 14.3% (7/49) | ＞0.05 |
| A4824G | ND2 | G | T-A | 6.3% | 6.4% (3/47) | 6.1% (3/49) | ＞0.05 |
| A4833G | ND2 | G | T-A | 6.3% | 8.5% (4/47) | 4.1% (2/49) | ＞0.05 |
| C4883T | ND2 | T |  | 21.9% | 23.4% (11/47) | 20.4% (10/49) | ＞0.05 |
| T5108C | ND2 | C |  | 7.3% | 10.6% (5/47) | 4.1% (2/49) | ＞0.05 |
| C5178A | ND2 | A | L-M | 20.8% | 23.4% (11/47) | 18.4% (9/49) | ＞0.05 |
| A5301G | ND2 | G | I-V | 6.3% | 6.4% (3/47) | 6.1% (3/49) | ＞0.05 |
| A5351G | ND2 | G |  | 5.2% | 4.3% (2/47) | 6.1% (3/49) | ＞0.05 |
| G5460A | ND2 | A | A-T | 5.2% | 2.1% (1/47) | 8.2% (4/49) | ＞0.05 |
| T5465C | ND2 | C |  | 5.2% | 4.3% (2/47) | 6.1% (3/49) | ＞0.05 |
| C5601T | tRNA | T |  | 6.3% | 8.5% (4/47) | 4.1% (2/49) | ＞0.05 |
| G6179A | COX1 | A |  | 6.3% | 4.3% (2/47) | 8.2% (4/49) | ＞0.05 |
| T6392C | COX1 | C |  | 18.8% | 21.3% (10/47) | 16.3% (8/49) | ＞0.05 |
| C6455T | COX1 | T |  | 7.3% | 6.4% (3/47) | 8.2% (4/49) | ＞0.05 |
| G6962A | COX1 | A |  | 13.5% | 17.0% (8/47) | 10.2% (5/49) | ＞0.05 |
| C7196A | COX1 | A |  | 12.5% | 10.6% (5/47) | 14.3% (7/49) | ＞0.05 |
| A7828G | COX2 | G |  | 5.2% | 6.4% (3/47) | 4.1% (2/49) | ＞0.05 |
| C8414T | ATP8 | T | L-F | 14.6% | 17.0% (8/47) | 12.2% (6/49) | ＞0.05 |
| G8584A | ATP6 | A | A-T | 16.7% | 12.8% (6/47) | 20.4% (10/49) | ＞0.05 |
| C8684T | ATP6 | T | T-I | 6.3% | 4.3% (2/47) | 8.2% (4/49) | ＞0.05 |
| A8701G | ATP6 | G | T-A | 56.3% | 61.7% (29/47) | 51.0% (25/49) | ＞0.05 |
| C8794T | ATP6 | T | H-Y | 5.2% | 6.4% (3/47) | 4.1% (2/49) | ＞0.05 |
| G9053A | ATP6 | A | S-N | 8.3% | 10.6% (5/47) | 6.1% (3/49) | ＞0.05 |
| G9123A | ATP6 | A |  | 6.3% | 6.4% (3/47) | 6.1% (3/49) | ＞0.05 |
| T9540C | COX3 | C |  | 55.2% | 61.7% (29/47) | 49.0% (24/49) | ＞0.05 |
| G9548A | COX3 | A |  | 6.3% | 8.5% (4/47) | 4.1% (2/49) | ＞0.05 |
| T9824C | COX3 | C |  | 7.3% | 6.4% (3/47) | 8.2% (4/49) | ＞0.05 |
| G10310A | ND3 | A |  | 18.8% | 21.3% (10/47) | 16.3% (8/49) | ＞0.05 |
| A10397G | ND3 | G |  | 8.3% | 8.5% (4/47) | 8.2% (4/49) | ＞0.05 |
| A10398G | ND3 | G | T-A | 62.5% | 66.0% (31/47) | 59.2% (29/49) | ＞0.05 |
| C10400T | ND3 | T |  | 56.3% | 61.7% (29/47) | 51.0% (25/49) | ＞0.05 |
| T10535C | ND4L | C |  | 5.2% | 6.4% (3/47) | 4.1% (2/49) | ＞0.05 |
| G10586A | ND4L | A |  | 6.3% | 6.4% (3/47) | 6.1% (3/49) | ＞0.05 |
| T10609C | ND4L | C | M-T | 12.5% | 14.9% (7/47) | 10.2% (5/49) | ＞0.05 |
| T10873C | ND4 | C |  | 56.3% | 61.7% (29/47) | 51.0% (25/49) | ＞0.05 |
| G11696A | ND4 | A | V-I | 5.2% | 8.5% (4/47) | 2.0% (1/49) | ＞0.05 |
| G11914A | ND4 | A |  | 6.3% | 4.3% (2/47) | 8.2% (4/49) | ＞0.05 |
| T11944C | ND4 | C |  | 6.3% | 6.4% (3/47) | 6.1% (3/49) | ＞0.05 |
| A12026G | ND4 | G | I-V | 6.3% | 6.4% (3/47) | 6.1% (3/49) | ＞0.05 |
| T12338C | ND5 | C | M-T | 5.2% | 6.4% (3/47) | 4.1% (2/49) | ＞0.05 |
| G12406A | ND5 | A | V-I | 12.5% | 14.9% (7/47) | 10.2% (5/49) | ＞0.05 |
| C12705T | ND5 | T |  | 62.5% | 70.2% (33/47) | 55.1% (27/49) | ＞0.05 |
| C12882T | ND5 | T |  | 12.5% | 14.9% (7/47) | 10.2% (5/49) | ＞0.05 |
| A13563G | ND5 | G |  | 6.3% | 8.5% (4/47) | 4.1% (2/49) | ＞0.05 |
| G13708A | ND5 | A | A-T | 5.2% | 6.4% (3/47) | 4.1% (2/49) | ＞0.05 |
| G13759A | ND5 | A | A-T | 9.4% | 10.6% (5/47) | 8.2% (4/49) | ＞0.05 |
| G13928C | ND5 | C | S-T | 20.8% | 21.3% (10/47) | 20.4% (10/49) | ＞0.05 |
| T14470C | ND6 | C |  | 7.3% | 6.4% (3/47) | 8.2% (4/49) | ＞0.05 |
| G14569A | ND6 | A |  | 8.3% | 10.6% (5/47) | 6.1% (3/49) | ＞0.05 |
| C14668T | ND6 | T |  | 14.6% | 17.0% (8/47) | 12.2% (6/49) | ＞0.05 |
| T14783C | Cytb | C |  | 56.3% | 61.7% (29/47) | 51.0% (25/49) | ＞0.05 |
| G15043A | Cytb | A |  | 56.3% | 61.7% (29/47) | 51.0% (25/49) | ＞0.05 |
| G15301A | Cytb | A |  | 57.3% | 63.8% (30/47) | 51.0% (25/49) | ＞0.05 |
| A15487T | Cytb | T |  | 12.5% | 10.6% (5/47) | 14.3% (7/49) | ＞0.05 |
